# Enhancement of the Acrolein-Induced Production of Reactive Oxygen Species and Lung Injury by GADD34

**DOI:** 10.1155/2015/170309

**Published:** 2015-03-03

**Authors:** Yang Sun, Sachiko Ito, Naomi Nishio, Yuriko Tanaka, Nana Chen, Lintao Liu, Ken-ichi Isobe

**Affiliations:** ^1^Department of Immunology, Nagoya University Graduate School of Medicine, Nagoya 466-8550, Japan; ^2^Department of Immunology, Norman Bethune College of Medicine, Jilin University, Changchun 130021, China

## Abstract

Chronic obstructive pulmonary disease (COPD) is characterized by lung destruction and inflammation. As a major compound of cigarette smoke, acrolein plays a critical role in the induction of respiratory diseases. GADD34 is known as a growth arrest and DNA damage-related gene, which can be overexpressed in adverse environmental conditions. Here we investigated the effects of GADD34 on acrolein-induced lung injury. The intranasal exposure of acrolein induced the expression of GADD34, developing the pulmonary damage with inflammation and increase of reactive oxygen species (ROS). Conversely, the integrality of pulmonary structure was preserved and the generation of ROS was reduced in GADD34-knockout mice. Acrolein-induced phosphorylation of eIF2*α* in GADD34-knockout epithelial cells by shRNA protected cell death by reducing misfolded protein-caused oxidative stress. These data indicate that GADD34 participates in the development of acrolein-induced lung injury.

## 1. Introduction

Acrolein is a reactive *α*, *β*-unsaturated aldehyde, which is abundant in organic combustion such as cigarette smoke, automobile exhaust, and manufacturing and cooking emission [1, 2]. Acrolein contains an active carbonyl group, which is highly reactive as biomacromolecule, and induces lung diseases, such as COPD [3], chronic bronchitis, emphysema [4], and lung carcinogenesis [5]. Acrolein has been shown to induce G to T and G to A mutations forming mutagenic a- and c-hydroxy-1, N (2)-cyclic propano-2′-deoxyguanosine adducts, which enhance oxidative DNA damage-induced mutagenesis [6]. Hochman et al. showed that acrolein produced reactive oxygen species (ROS) in mast cells [7].

Recently we have demonstrated that acrolein can induce pulmonary injury and macrophage infiltration. Beyond that, acrolein can cause oxidative stress and generate reactive oxygen species (ROS) [8]. Although the mechanisms are incompletely understood, ROS generation occurs in a wide variety of human diseases. The endoplasmic reticulum (ER) is a key organelle, where proteins are newly synthesized. When the newly synthesized proteins are misfolded, these proteins accumulate and cause ER stresses or unfolded protein response (UPR). There exist three main ER stress responses in mammals. First, PERK-eIF2*α* pathway attenuates translation to limit the accumulation of unfolded proteins. Second, activation of transcription factor 6 (ATF6) induces chaperones such as glucose regulated protein-78 (Grp78)/immunoglobulin binding protein (BiP). Third, Inositol-requiring enzyme 1 (IRE1) cuts the precursor XBP1 mRNA twice, removing an internal fragment and thus inducing a frame shift. ER stress responses provide a conserved mechanism by reducing the folded protein load (eIF2*α* phosphorylation and ERAD degradation) and increase the folding capacity (induction of Bip/GRP78) [9, 10]. It has been shown that acrolein induces these three UPRs [11–14].

Growth arrest and DNA damage-inducible protein (GADD34/Ppp1r15a) was originally isolated from UV-inducible transcripts in Chinese hamster ovary (CHO) cells [15]. Expression of GADD34 is upregulated by growth arrest and DNA damage [16]. It is also induced by amino acid deprivation and several endoplasmic reticulum (ER) stresses [17, 18]. GADD34 dephosphorylates several kinases that function in important signaling cascades, including dephosphorylation of eIF2 [17]. Because acrolein is a DNA damaging agent and induces ER stresses [14] and induces myeloid infiltration to lung [19], here we investigated whether GADD34 might affects pathogenesis of acrolein-induced lung injury.

## 2. Materials and Methods

### 2.1. Mice and Acrolein Administration

Eight-week-old female wild-type C57BL/6 mice were obtained from SLC Japan (Shizuoka, Japan). GADD34-knockout mice were generated as previously described [20]. All mice were maintained in pathogen-free facilities in the Animal Research Center at the Nagoya University Graduate School of Medicine. They were maintained at 25°C with a 55% relative humidity and a 12 h light-dark cycle.

For the lung injury studies, mice were randomly allocated into 3 groups (*n* = 6, per group). The mice were instilled intranasally with acrolein (5 *μ*mol/kg, Sigma); control mice received equal volume of phosphate buffer saline (PBS, pH = 7.4) alone. The mice were treated daily for 5 d/week for up to 28 days. The mice were sacrificed at 7 and 28 days; then, the lungs were isolated and used for histological analysis and/or flow cytometry analysis.

### 2.2. Cell Culture

Murine bone marrow-derived macrophage cells were established previously by Ito et al. [21]. The cells were cultured in RPM1640 (Sigma) with 10% fetal bovine serum (FBS; Gibco, Grand Island, NY) and 10% GM-CSF which was produced by murine GMCSF-producing Chinese hamster ovary (CHO) cells (GM-CSFCM).

Lewis lung carcinoma (LLC) cells were obtained from Riken [22]. Cells were cultured in HEPES-buffered Dulbecco's modified Eagle's medium (DMEM) containing 10% FBS (Hyclone).

### 2.3. Lentivirus-Mediated shRNA Knockdown of Gene Expression

The translation of GADD34 mRNA in LLC cells was knocked down using the Mission TRC mouse shRNA Lentivirus Transduction Particles (pLKO.1-puro, Sigma). The sequences of the shRNAs used were CCGGGGCGGCTCAGATTGTTCAAAGCTCGAGCTTTGAACAATCTGAGCCGCCTTTTTG (shRNA TRC2, targeting exon 2) for GADD34 knockdown. Nontarget control shRNAs (Sigma, SHC 202V) were used as a control. LLC cells were infected with viral particles and treated with 8 *μ*g/mL polybrane (Millipore TR-1003-G) and then incubated with cells for 24 h. Cells expressing shRNA were selected on 2 *μ*g/mL puromycin (Sigma, P8833) for functional studies. The extent of knockdown of GADD34 expression was confirmed by real-time PCR. Recombinant experiments used here were approved by Committee of Nagoya University Graduate School of Medicine. Established GADD34 knockdown LLCs (shGAD34/LLCs) and control LLCs (shcon/LLCs) were used.

### 2.4. Histology and Immunohistochemistry

For histological analysis, the lung tissue were isolated from mice after acrolein treatment, and 5 *μ*m frozen lung sections were stained with hematoxylin and eosin (H&E). For immunofluorescence analysis, cryosections were fixed in cold acetone and blockade with 2% BSA-PBS for 1 h; sections were incubated with rabbit anti-ProSpC (epithelial type II cells marker) at 4°C, overnight. The sections were stained with Alexa Flour 448 goat anti-rabbit IgG for 1 h and counterstaining with DAPI for 5 min.

### 2.5. Flow Cytometry

The whole lung was minced in the cold PBS. After centrifugation, the tissue homogenate was suspended with Tris-NH_4_Cl red blood cells (RBCs) lysing buffer (150 mM sodium chloride, 1% Triton, 0.5% sodium deoxycholate, 0.1% SDS, 50 mM Tris) to lysed RBCs. Cells (1 × 10^5^/sample) were blocked in 50 *μ*L 0.2% BSA-PBS, stained with FITC-conjugated anti-Gr1, PE-conjugated anti-CD11b, anti-CD11c, and APC-conjugated anti-F4/80 (BD Biosciences) for 30 min at 4°C, and analyzed by flow cytometry using a FACS Canto flow cytometer (BD Biosciences).

### 2.6. ELISA

Cells (GM-IMs) were seeded in 12-well plates (3 × 10^5^/well) and treated with 10 *μ*M acrolein (Sigma) for 12 and 24 h. The level of IL-6 in culture medium was measured by ELISA kits, according to the manufacturer's instructions (R&D systems).

### 2.7. ROS Measurements and Inhibition of ROS Production

Lung tissue was isolated from mouse and minced; after lysed RBCs, cells (1 × 10^5^/well) were seeded in 12-well plates, stained with 2 *μ*M carboxy-2′,7′-dihydrofluorescein diacetate (H2DCFDA) (Invitrogen) for 30 min at 37°C. The cells were collected and ROS generation was determined by flow cytometry.

The shcon/LLCs and shGADD34/LLCs were plated in 12-well dishes and treated with 25 *μ*M acrolein alone for 8 h, or 25 *μ*M acrolein with 10 *μ*M MG132 or/and 1 *μ*g/mL cycloheximide (CHX) for 12 h. After washing, cells were incubated with 2 *μ*M H2DCFDA for 30 min at 37°C. Collected cells were analyzed for ROS generation by flow cytometry. ROS inhibitor N-acetyl-L-cysteine (NAC) (Sigma-Aldrich) was dissolved at 1 mol/L in deionized water.

### 2.8. Western Blotting

Lung tissue and cells RIPA lysis buffer (0.1 M PBS, pH 7.4 containing 1% deoxycholic acid sodium, 0.2% SDS, and protease inhibitors). After measurement of protein concentration, the samples were loaded and separated by sodium dodecylsulfate polyacrylamide gel electrophoresis (SDS-PAGE) and then transferred to Immobilon transfer membranes. The membranes were incubated with primary anti-phospho-NF-*κ*B P65 (Ser536) antibody, anti-caspase 3, and anti-p-eIF2*α* (Cell Signaling), anti-GADD153, and anti-GADD34 (Santa Cruz Biotechnology) overnight at 4°C. Then, the membranes were incubated with the secondary anti-rabbit IgG for 1 h. Blots were developed with western blot detection reagent (GE Healthcare).

### 2.9. Real-Time PCR

Total RNA was extracted with TRIzol Reagent and reverse-transcribed using a High Capacity cDNA Reverse Transcription Kit (Applied Biosystems). Quantitative real-time PCR was performed using the MX3000P QPCR System (Agilent) according to the manufacturer's protocol. The sequences of the RT-PCR primers for each pair are listed in Table 1.

### 2.10. Apoptosis

The shcon/LLCs and shGADD34/LLCs were plated in 12-well dishes and treated with 25 *μ*M acrolein alone for 8 h, or 25 *μ*M acrolein with 10 *μ*M MG132 or/and 1 *μ*g/mL cycloheximide (CHX) for 12 h. Cells were collected and with FITC-labeled annexin V and 7-AAD (BD Bioscience) for 15 min. Cells were analyzed by flow cytometry on a FACS Calibur. Data were analyzed by FlowJo software (TreeStar).

### 2.11. Protein Synthesis Analysis

Shcon/LLCs and shGADD34/LLCs were collected after treatment. To count the cell number, samples were stained with Trypan Blue. We centrifuge the cell in 400 rpm for 3 min, and the supernatant were thrown away. An equal number of live cells were extracted with RIPA lysis buffer (2 × 10^5^ cells/100 *μ*L). All samples were analyzed by SDS-PAGE.

### 2.12. Statistics

Data are expressed as means ± standard error of the mean (s.e.m.). Statistical comparisons were performed by ANOVA followed by Fisher's post hoc test. Values of *P* < 0.05 were considered statistically significant.

## 3. Results

### 3.1. NAC Prevents Acrolein-Induced Lung Injury and Inflammation

**Figure 1:**
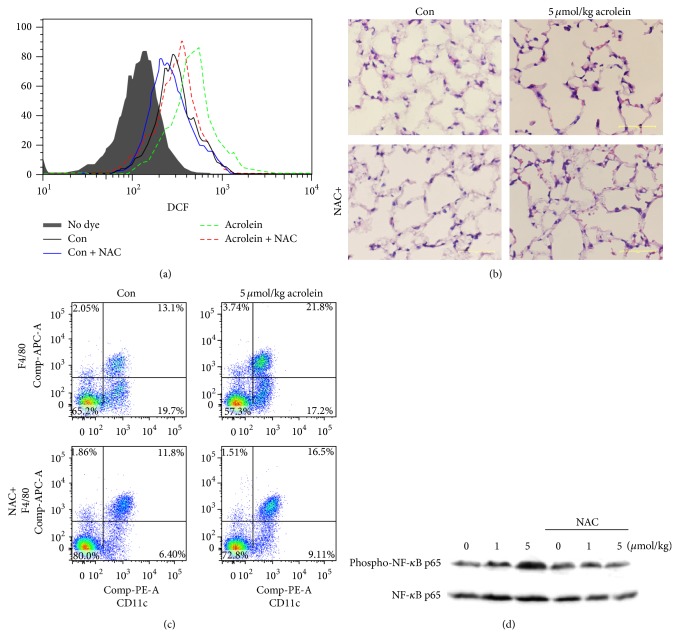
NAC prevents acrolein-induced lung injury and inflammation. Wild-type mice were intranasally instilled by 5 *μ*mol/kg acrolein with intravenous injection of 100 *μ*L of NAC (500 mg/kg) or equal volume of PBS. (a) Level of ROS in lung tissues was measured by DCFH-DA. (b) H&E staining of lung tissues (scale bar: 50 *μ*m). (c) Alveolar macrophages were stained with PE-conjugated anti-CD11c and APC-conjugated anti-F4/80 and detected by FACS. (d) The expression of phospho-NF-*κ*B p65 in the lung tissues by western blot.

ROS generation impaired oxidant defense contributes to the organ injury [23]. We found that ROS was greatly produced from the lung at day 7 and was decreased by antioxidant NAC treatment (Figure 1(a)). On the basis of analysis of lung sections stained with H&E staining, NAC prevented alveolar damage and immune cell migration, which were caused by acrolein in lung tissue (Figure 1(b)). F4/80^high^ CD11c^+^ alveolar macrophages were recruited to the site of injury (Figure 1(c)). It has been shown that smoke-associated oxidative stress may promote lung inflammation through NF-*κ*B signaling [24]. We also observed the expression of phospho-NF-*κ*B p65 by acrolein (7d), which was completely abolished by NAC (Figure 1(d)).

### 3.2. GADD34 Mediates Acrolein-Caused Lung Injury

**Figure 2:**
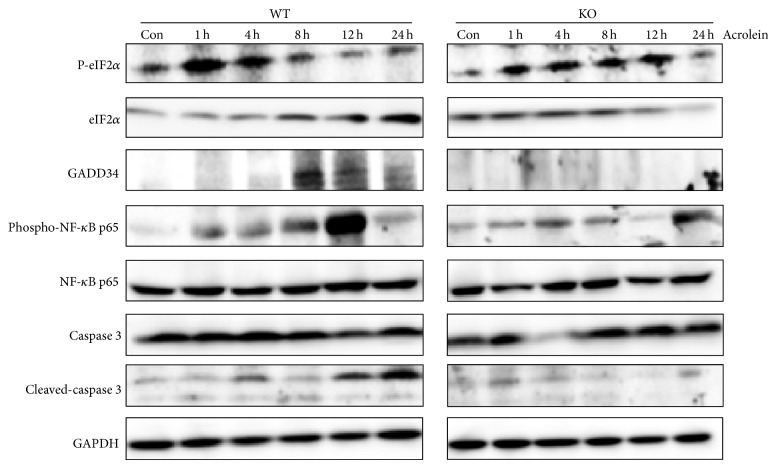
Acrolein activates ER stress in lung tissue. Protein expression of ER stress was analyzed. Wild-type GADD34-knockout mice were intranasally instilled by 5 *μ*mol/kg acrolein. Lung tissues were collected at the indicated times for western blot analysis.

Acrolein has been shown to induce ER stress [14]. We examined the expression of ER stress markers in lung tissue of wild-type and GADD34-knockout mice after nasal injection of acrolein (5 *μ*mol/kg) (Figure 2). In wild-type mice, the expression of p-eIF2*α* increased highly between 1 and 4 h after acrolein exposure, which is followed by induction of GADD34; then, p-eIF2*α* expression was decreased from 8 h. After ER stress, we observed the increase of cleaved caspase 3, which might cause lung tissue destruction. However, GADD34-knockout mice continuously express higher level of p-eIF2*α*; the cleaved caspase 3 level was lower than that in wild-type mice.

**Figure 3:**
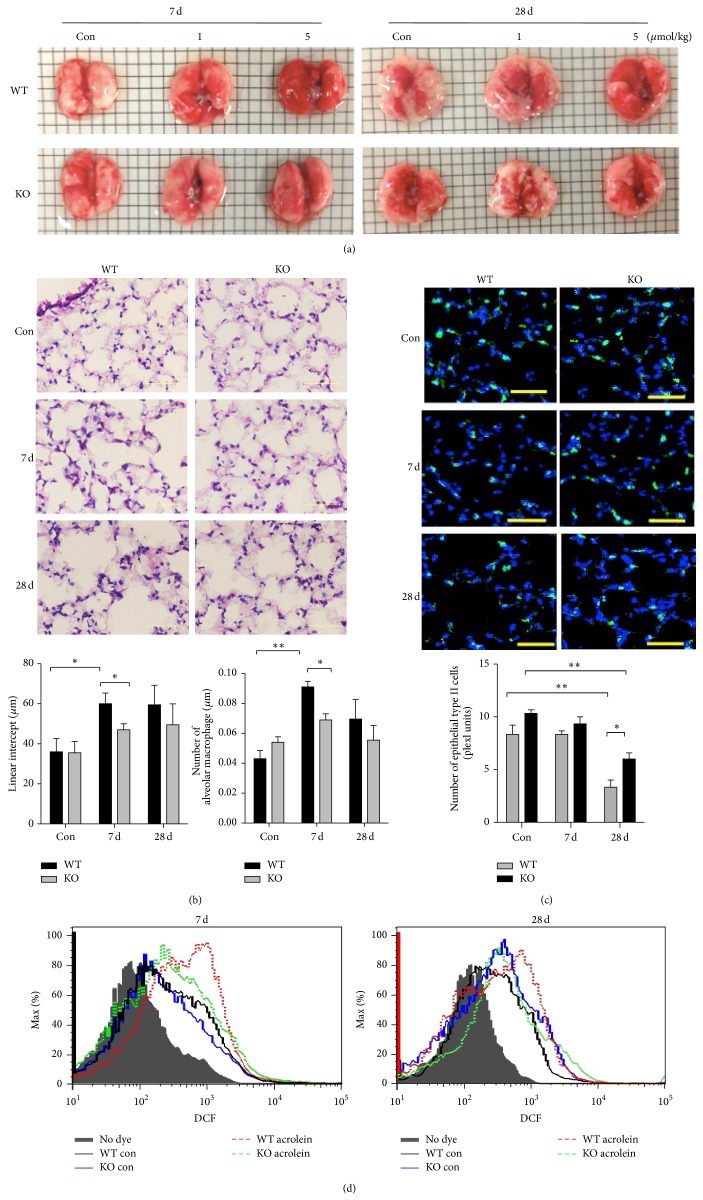
GADD34 mediates acrolein-caused lung injury. The wild-type and GADD34-knockout mice were intranasally instilled by 1 and 5 *μ*mol/kg acrolein. The mice were treated daily for 5 d/week for up to 28 days and then were sacrificed at 7 and 28 days. (a) The whole lungs of wild-type and GADD34-knockout mice were photographed at days 7 and 28. (b) H&E staining of lung tissue. Scale bar: 50 *μ*m. Analysis of alveolar length determined by mean linear intercepts (*n* = 5 to 7 mice in each group) and the number of alveolar macrophages in wild-type and GADD34-knockout mice (*n* = 4 mice in each group). (c) Lungs stained for epithelial type II cells (ProSpC green), nuclei (DAPI blue), and the number of epithelial type II-positive cells in wild-type and GADD34-knockout mice (10 fields, *n* = 4). Scale bar: 50 *μ*m. ^*^ *P* < 0.05, ^**^ *P* < 0.01. Data are represented as means ± s.e.m. (d) Levels of ROS production in the lung of wild-type and GADD34-knockout mice were measured by DCFH-DA after acrolein treated.

Our previous studies have demonstrated that intranasal instillation of acrolein (5 *μ*mol/kg) induced lung damage and hemorrhage [8]. We examined whether GADD34-knockout mice showed same phenotypes by intranasal instillation of acrolein. Although acrolein administration caused serious alveolar structure destruction, such as airspace enlargement, and hemorrhaging in the lung of wild-type mice, GADD34 deficiency decreased the lung injury with preserved alveolar structure, no significant hemorrhaging, and sparse accumulation of intra-alveolar macrophages at 7 and 28 days (Figures 3(a) and 3(b)). The numbers of type II epithelial cells were significantly reduced in wild-type mice compared to GADD34-knockout mice (Figure 3(c)). In addition, higher level of ROS production was detected in wild-type mice than GADD34-knockout mice (Figure 3(d)). These results collectively indicated that GADD34 might play a crucial role in the pathogenesis of experimental acrolein-induced pulmonary injury.

### 3.3. Low Level of Pulmonary Inflammation in GADD34-Knockout Mice Induced by Acrolein

**Figure 4:**
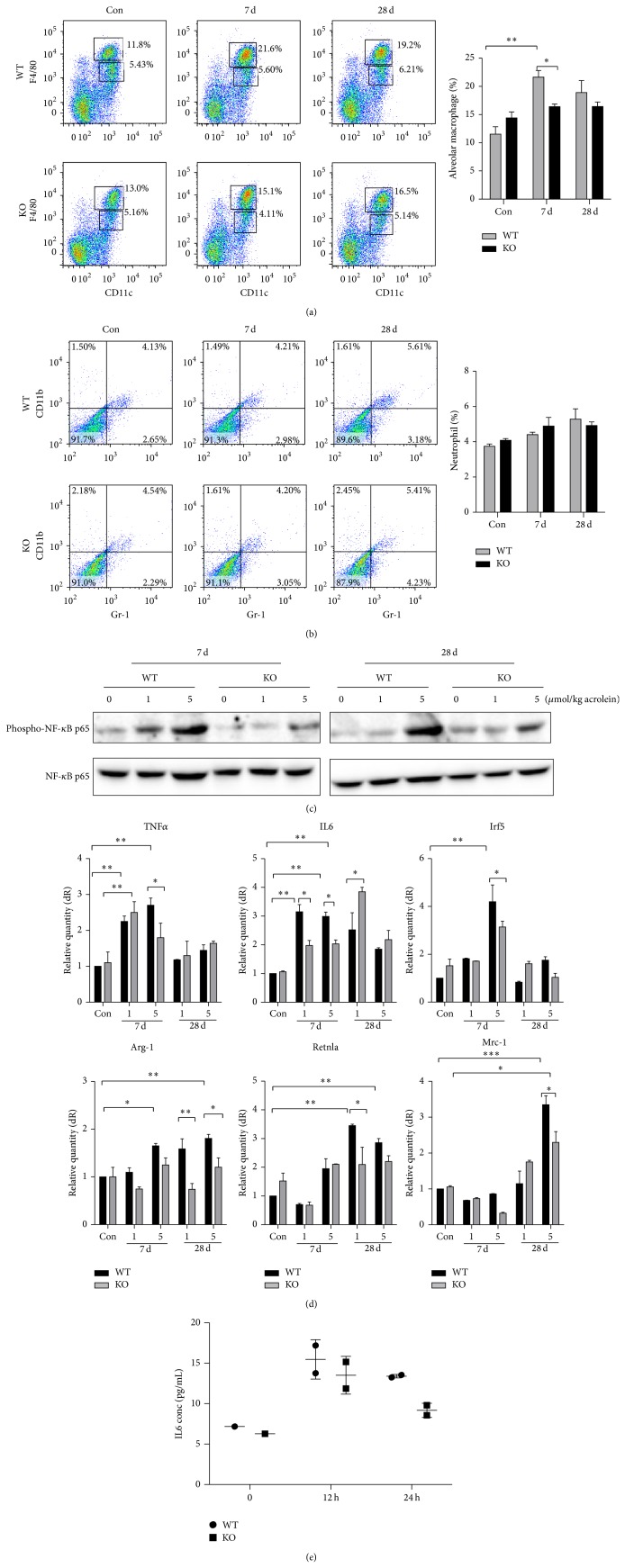
Low level of pulmonary inflammation in GADD34-knockout mice induced by acrolein. Lungs were collected from wild-type and GADD34-knockout mice at days 7 and 28 after 5 *μ*mol/kg acrolein instillation. (a) Alveolar macrophages as F4/80^high^CD11c^+^ and (b) neutrophils as Gr-1^+^CD11b^+^ were confirmed by FACS. (c) The expression of phospho-NF-*κ*B p65. (d) The expressions of macrophage type I markers, TNF*α*, IL-6, and Irf5, and macrophage type II markers, Arg-1, Mrc-2, and Retnla, were analyzed by quantitative real-time PCR. (e) Wild-type and GADD34-knockout mice macrophages were cultured in 12-well plastic plates and stimulated with 10 *μ*M acrolein for 12 and 24 h. Supernatants were taken and IL-6 expression was analyzed by ELISA. Data shown are the mean ratios ± SE of three separate experiments. Data are represented as means ± s.e.m. ^*^ *P* < 0.05, ^**^ *P* < 0.01.

We have demonstrated that the acrolein-induced lung injury is accompanied by inflammatory response. To investigate whether GADD34 affects the pathologies of pulmonary inflammatory responses, the mice were instilled with acrolein for 7 or 28 days to generate acute inflammation. The increase of F4/80^high^CD11c^+^ macrophages was lower in GADD34-knockout mice than those in wild-type mice (Figure 4(a)). However, the GR-1^+^CD11b^+^ neutrophils migration was not observed at 7 or 28 days after acrolein treatment both in wild-type and in GADD34-knockout mice. (Figure 4(b)). Acrolein-induced lung damage may promote lung inflammation through NF-*κ*B signaling. A sizable NF-*κ*B response with phosphorylation of p65 on Ser536 was observed in wild-type mice, whereas this response was lowered in GADD34-knockout mice (Figure 4(c)).

To assess the gene expression levels of inflammatory cytokine, the real-time PCR (RT-PCR) was performed. GADD34-knockout mice showed significantly lower levels of M1 macrophages including TNF*α*, IL-6, and Irf5 at 7 days compared with wild-type mice. The expression of TNF*α* and Irf5 decreased at 28 days in both wild-type and GADD34-knockout mice. In addition, only at late time (at 28 days after acrolein administration) wild-type mice expressed high level of M2 macrophage markers such as Arg1 and Mrc-1. These expressions were lower in GADD34-knockout mice (Figure 4(d)). Then we examined the protein expression of IL-6 in supernatant of wild-type and GADD34-knockout macrophage cell line. By the stimulation of acrolein IL-6 protein expression was higher in wild-type macrophages than that in GADD34-knockout macrophages (Figure 4(e)).

### 3.4. GADD34 Is a Mediator on ER Stress-Induced Oxidative Stress

**Figure 5:**
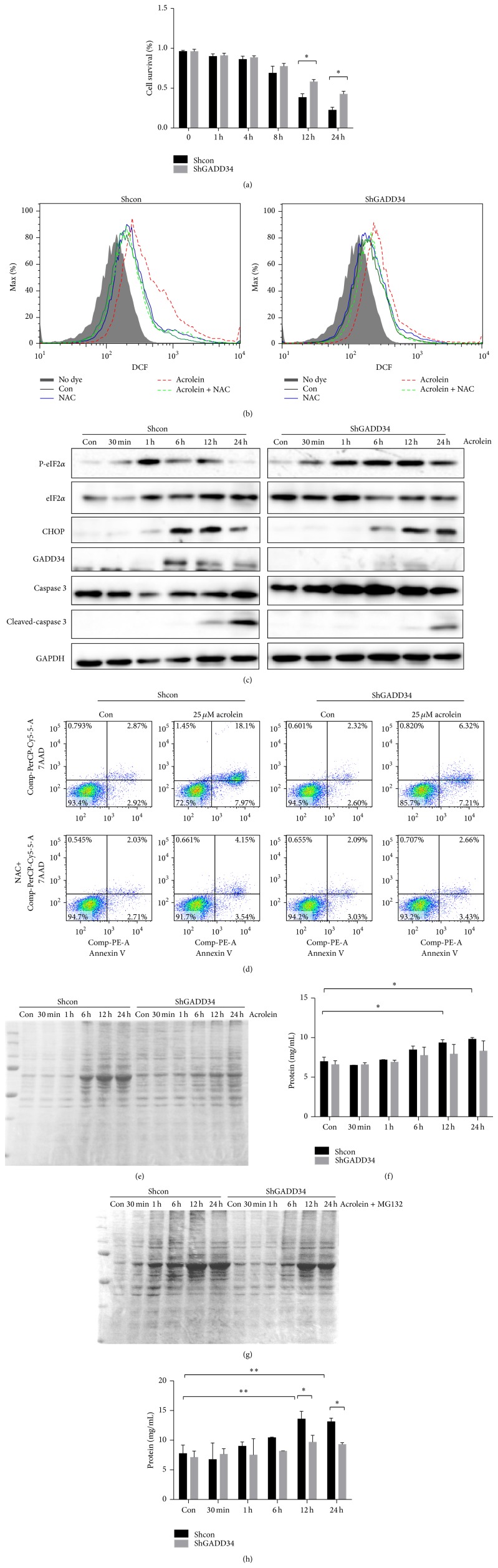
GADD34 promote recovery from a shutoff of total protein synthesis and enhance cell death. The 25 *μ*M acrolein-treated shcon/LLCs and shGADD34/LLCs were analyzed. (a) Cell survival after 25 *μ*M acrolein treatment was measured. ^*^ *P* < 0.05. (b) Levels of ROS production in shcon/LLCs and shGADD34/LLCs were measured by DCFH-DA after 25 *μ*M acrolein treated for 8 h. (c) Cells were collected at the indicated times and protein expressions of ER stress signaling were detected by western blot. (d) The 25 *μ*M acrolein-treated shcon/LLCs and shGADD34/LLCs with 20 mM NAC or without NAC for 8 h. Cells were stained with Annexin V-PE/7-amino-actinomycin D (7AAD). (e) Bands of proteins were analyzed by SDS-PAGE after 25 *μ*M acrolein treatment. (f) Protein concentration was measured by Bio-Rad Protein Assay. (g) The shcon/LLCs and shGADD34/LLCs were treated by 25 *μ*M acrolein with 10 *μ*M MG132. Cells were collected and lysate was analyzed by SDS-PAGE. (h) Amount of proteins was measured by Bio-Rad Protein Assay. Data are represented as means ± s.e.m. ^*^ *P* < 0.05.

In order to understand the molecular mechanisms of effects of GADD34 on acrolein-induced lung injury, we used lung cell line LLCs. ShGADD34/LLCs died later than shcon/LLCs by the stimulation of 25 *μ*M acrolein (Figure 5(a)). A large amount of ROS was produced from shcon/LLCs by the acrolein treatment, which was blocked by NAC. ROS production was attenuated by GADD34 deficiency (Figure 5(b)). Because ROS was produced highly in shcon/LLC by acrolein administration compared to that in shGADD34/LLC, cleaved caspase 3 was highly increased in shcon/LLC at 24 h (Figure 5(c)). Then we examined the effects of ER stresses induced by acrolein. In shcon/LLCs, the expression of p-eIF2*α* increased early and then decreased by acrolein treatment in shcon/LLC. Expression of CHOP was increased after the early increase of p-eIF2*α* in shcon/LLCs, which induced GADD34; then GADD34 dephosphorylated eIF2*α*. In shGADD34/LLC, the expression of p-eIF2*α* continued to be expressed, because of the absence of GADD34.

Subsequently, the execution phase of apoptosis was analyzed to determine the effect of acrolein on shcon and shGADD34 LLCs. There was a significant increase in annexin V, 7-AAD double positive cells in shcon/LLCs after 8 h exposure of acrolein compared with shGADD34/LLCs (Figure 5(d)).

### 3.5. GADD34 Promote Recovery from a Shutoff of Total Protein Synthesis and Enhance Cell Death

In fact, the extracellular stimuli and changes in intracellular homeostasis cause protein misfolding in the endoplasmic reticulum. ER stress caused unfolded protein response (UPR) is a cellular adaptive response that evolved to restore protein-folding homeostasis by reducing protein synthesis. Phosphorylation of eIF2*α* limits initiation of translation on many cellular mRNAs within the cells. To clarify whether strongly phosphorylated eIF2*α* in shGADD34/LLCs can affect translation of related protein, the protein synthesis was examined. We found that proteins in acrolein-treated WT cells (shcon/LLCs) do not increase early phase by shutoff of protein synthesis caused by p-eIF2*α*. Protein synthesis gradually recovered in shcon/LLCs after 6 h but remained at lower levels in shGADD34/LLCs (Figures 5(e) and 5(f)).

**Figure 6:**
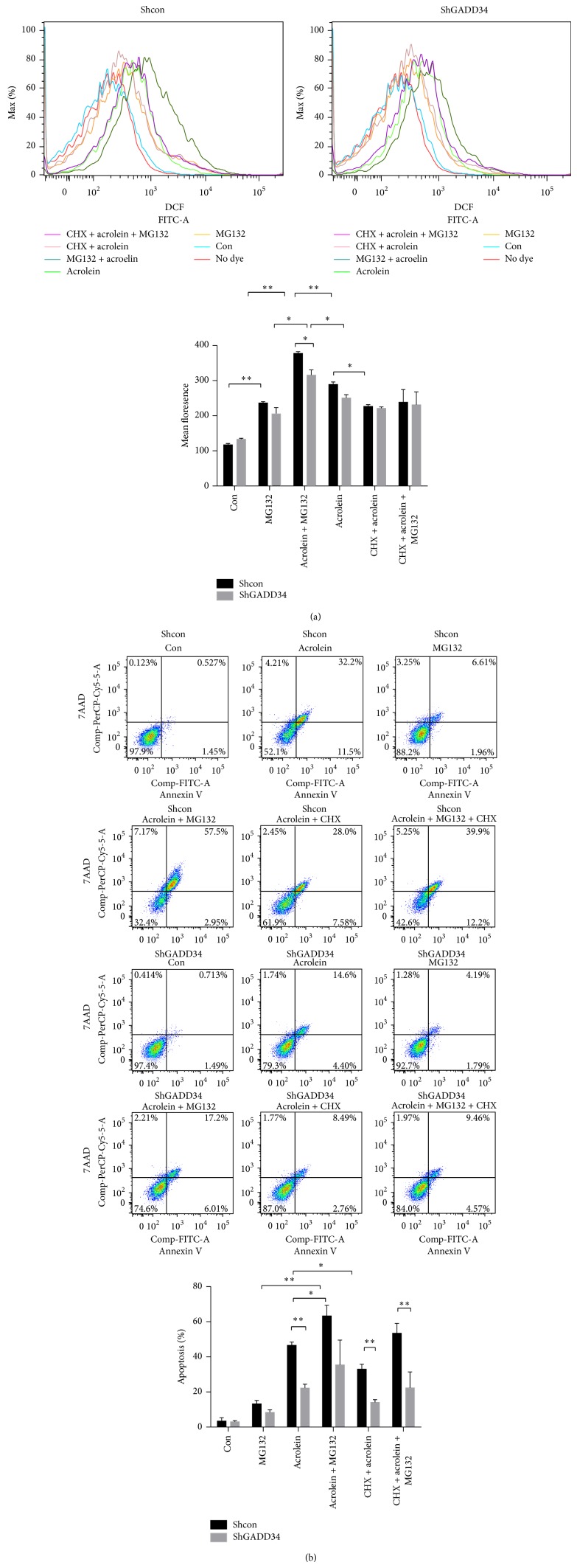
Protein synthesis promotes ROS production and cell death. The shcon/LLCs and shGADD34/LLCs were treated by 25 *μ*M acrolein with 10 *μ*M MG132 and/or 1 *μ*g/mL CHX or without these agents for 12 h. (a) ROS levels were measured by DCFH-DA fluorescence by flow cytometry. Right, mean fluorescence after subtracting autofluorescence. ^*^ *P* < 0.05, ^**^ *P* < 0.01. (b) Cells were stained with Annexin V-PE/7-AAD and then analyzed by flow cytometry. ^*^ *P* < 0.05, ^**^ *P* < 0.01.

Ubiquitin-proteasome system controls the degradation of a large number of cellular proteins including short-lived, regulatory, and damaged or misfolded protein [25, 26]. It has been assumed that accumulation of no longer needed proteins underlies the toxicity of proteasome inhibition. Proteasome inhibition can induce the integrated stress response (ISR) [27]. The ISR is an adaptive response to many forms of stresses, which converge into phosphorylation of eIF2*α* [28]. We examined whether inhibition of the proteasome enhanced misfolded proteins synthesis by the addition of MG132 to acrolein. We found that the protein expression was enhanced in later time in both shcon/LLCs and shGADD34 by the addition of MG132. However, the level of protein expression was less in shGADD34/LLCs than that in shcon/LLCs by the addition of MG132 (Figures 5(g) and 5(h)). These results confirmed that protein synthesis was decreased by the expression of GADD34. We also discovered that acrolein induced higher level of ROS production and cell death in shcon/LLCs, although these treatments had less effects on ROS production of shGADD34/LLCs (Figures 6(a) and 6(b)). The addition of MG132 to acrolein treatment enhanced ROS generation and cell death in shcon/LLCs. Protein synthesis may play a pivotal role in ROS production and cell death [29, 30]. A protein synthesis inhibitor (CHX) was added to the treatment of acrolein. CHX inhibited the generation of ROS and cell death in both shcon/LLCs and shGADD34/LLCs. But suppressions of ROS production and cell death were higher in shcon/LLCs than that in shGADD34/LLCs (Figures 6(a) and 6(b)). These results indicated that highly phosphorylated eIF2*α* in shGADD34/LLCs led to a reduction of synthesized protein, which decreased the ROS-induced cell death.

## 4. Discussion

COPD is characterized by chronic inflammation and destruction of the lung [31]. It is a major clinical challenge mostly due to cigarette smoke exposure [32]. Our findings indicated that acrolein not only caused pulmonary structure damage, but also promoted pulmonary inflammation through NF-*κ*B signaling* in vivo*. Alveolar injury caused by acrolein exposure might be relevant to cigarette smoke-induced chronic lung destruction.

It has been shown that ER stresses induce cell death by CHOP following PERK-mediated eIF2*α* phosphorylation [24, 33]. We examined whether acrolein induces eIF2*α* phosphorylation and CHOP expression* in vitro*. We showed that CHOP was strongly expressed by the treatment of acrolein in shcon/LLC. In shGADD34/LLC expression of p-eIF2*α* was also increased by acrolein treatment. In contrast to shcon/LLC, expression of p-eIF2*α* in shGADD34/LLC continued long time, because of the lack of GADD34 expression. Continued expression of p-eIF2*α* in shGADD34/LLC induced the later expression of CHOP in these cells. From these results cell death in shcon/LLCs is not caused by CHOP expression.

In LLC/shcon, the acrolein-induced protein synthesis was upregulated, which induced oxidative stress. Generated ROS caused cell death by upregulating caspase 3. In contrast, the continuous expressions of p-eIF2*α* in shGADD34/LLC cells shut off the synthesis of protein, which caused the remission of oxidative stress in shGADD34/LLCs. We used proteasome and protein synthesis inhibitors to support our results. MG132 is a proteasome inhibitor, which accumulates proteins. Accumulated proteins induce oxidative stress, which induce cell death. Because p-eIF2*α* induced by acrolein in shGADD34/LLCs was higher than that in shcon/LLCs, protein accumulation by MG132 was higher in shGADD34/LLCs than that in shcon/LLCs. Further we showed that proteins in shcon/LLC were greatly decreased by the stimulation of acrolein, which reduced the formation of ROS as well as cell death. Oxidative stress is recognized as a major predisposing factor in the pathogenesis of COPD [34]. Alveolar macrophages from patients with COPD are more activated and release increased amount of ROS [35]. The endogenous oxidative stress is generated by mitochondria when the lung gets injury, and then the ROS induces strong inflammatory responses and severe damage in lung. Previous studies have confirmed that the generation of ROS contributes to bactericidal activity of macrophages [36]. Because we observed higher caspase 3 expression in shcon/LLCs, ROS might produce mitochondrial damages by direct acrolein exposure. It has been shown that exposure of acrolein leads to mitochondrial dysfunction, which induces accumulation of ROS [37]. We found that GADD34 was highly expressed under the stimulation of acrolein, indicating GADD34 might be involved in the pathogenesis of alveolar injury by producing ROS.

In conclusion, our results demonstrate that GADD34 is upregulated* in vivo* and* in vitro* by the exposure of acrolein. GADD34 reverts the phosphorylation of eIF2*α* induced by acrolein. Dephosphorylation of eIF2*α* accumulates misfolded proteins, which induces oxidative stress. Generated ROS from direct mitochondrial dysfunction by acrolein or ROS produced by ER stress induce cell death and macrophages infiltration. GADD34 is one of the key proteins in acrolein-induced lung inflammation and tissue injury.

## Figures and Tables

**Table 1:** 

Genes		Sequences (5′-3′)
*Irf5 *	Forward	GCTGGCTACAGGGTTCTGAG
	Reverse	CTGCTGGCTTCATTTCTTCC
*TNFα*	Forward	GCCCATATACCTGGGAGGAG
	Reverse	CACCCATTCCCTTCACAGAG
*IL6 *	Forward	CCGGAGAGGAGACTTCACAG
	Reverse	TCCACGATTTCCCAGAGAAC
*Arg1 *	Forward	GTGAAGAACCCACGGTCTGT
	Reverse	CTGGTTGTCAGGGGAGTGTT
*Retnla *	Forward	TGCTGGGATGACTGCTACTG
	Reverse	CTGGGTTCTCCACCTCTTCA
*Mrc1 *	Forward	CAAGGAAGGTTGGCATTTGT
	Reverse	CCTTTCAGTCCTTTGCAAGC
*Gapdh *	Forward	AACTTTGGCATTGTGGAAGG
	Reverse	ACACATTGGGGGTAGGAACA
